# Rearing of *Hyalomma marginatum* (Acarina: Ixodidae) under laboratory conditions in Morocco

**DOI:** 10.1007/s10493-021-00641-3

**Published:** 2021-07-06

**Authors:** Latifa Elhachimi, Félix Valcárcel, Angeles S. Olmeda, Sabrine Elasatey, Sarah E. Khattat, Sylvie Daminet, Hamid Sahibi, Luc Duchateau

**Affiliations:** 1 Département de Parasitologie et Santé Publique, Institut Agronomique et Vétérinaire Hassan II, B.P. 6202, Rabat, Morocco; 2 Grupo de Parasitología Animal, Animalario del Departamento de Reproducción Animal, INIA, 28040 Madrid, Spain; 3 Departamento de Sanidad Animal, Facultad de Veterinaria, UCM, 28040 Madrid, Spain; 4 Faculty of Veterinary Medicine, Ghent University, Salisburylaan 133, 9820 Merelbeke, Belgium

**Keywords:** *Hyalomma marginatum*, Morocco, Rearing, Life cycle time, Temperature

## Abstract

*Hyalomma marginatum* Koch is one of the main tick vectors of human and animal tick-borne diseases. The objective of this study was to establish standard procedures for rearing *H. marginatum* under laboratory conditions. Such laboratory tick populations are required to study acaricide resistance of *Hyalomma* ticks. In our rearing program, larvae and nymphs were fed on New Zealand white rabbits, whereas adults were fed on sheep. Non-parasitic stages were held at 18 and 28 °C to study the effect of temperature on development and survival. In our experiments, *H. marginatum* ticks have maintained the characteristics of a two-host life cycle. The engorged larvae did not detach and moulted on the rabbit, after which the emerged nymphs continued to feed on the same animal. The life cycle duration of *H. marginatum* was influenced by temperature, with each non-parasitic stage—i.e., larva and nymph molting—developing faster at 28 than at 18 °C; preoviposition and oviposition periods were shorter at 28 than at 18 °C. At 18 °C, no eggs hatched. The whole cycle from the collection of an engorged field tick until the emergence of second-generation larvae took 189 days. One such tick on average results in 3500 eggs which over time, taking into account the losses at each developmental stage, develop into 1200 adult ticks. Rearing these ticks a second generation therefore could result in millions of larval ticks.

## Introduction

Tick borne-diseases are a permanent threat to human and animal health worldwide. In Morocco, a total of 28 tick species including 15 hard ticks with seven species of the genus *Hyalomma* are widely distributed and known to transmit various diseases in cattle. *Hyalomma marginatum* Koch is widespread in various agro-climatic regions of Morocco (Bailly-Choumara et al. 1976; Ouhelli and Pandey 1982; Walker et al. 2003). It is a two-host tick with adults feeding on large mammals such as livestock and wild ungulates, whereas the larvae and nymphs feed on small mammals and ground-dwelling birds (Apanaskevich and Horak 2008; Santos-Silva and Vatansever 2017).

This tick species is very competent in the transmission of bovine tropical theileriosis caused mainly by *Theileria annulata* (Jongejan et al. 1983; Sayin et al. 2003) and piroplasmosis in Morocco (Seng et al. 2009; Hamou et al. 2012). It is also the main vector of the Crimean-Congo hemorrhagic fever virus to humans and the main vector for new emergences in previously uninfested regions (Zeller et al. 1994; Palomar et al. 2013; Chitima-Dobler et al. 2019). Rearing this ixodid tick under laboratory conditions is essential for investigating, amongst other goals, its phenotypic and genetic resistance against acaricides. Since the research conducted by Ouhelli (1994), no results have been published on the rearing of *Hyalomma* ticks under laboratory conditions in Morocco.

The objective of the present study is to investigate the rearing of *H. marginatum* under laboratory conditions and to establish standard procedures for producing *H. marginatum* laboratory colonies.

## Material and methods

### Tick collection

Ticks were collected from cattle in the Skhirat region (sub-humid) in July 2019. Healthy animals were randomly chosen at the collection site, and engorged female ticks were sampled from the udder, perineum, dewlap, ears, and neck. Immediately upon arrival at the laboratory, ticks were washed in distilled water, identified, weighed, put in filter paper and kept in individual tubes for egg-laying. The tick containers (5 × 2.5 cm) were covered with a muslin cloth and secured with a perforated lid. The identification of ticks was carried out under a stereo-microscope using the keys based on morpho-anatomical characteristics (Walker et al. 2003; Estrada-Peña et al. 2004; Apanaskevich and Horak 2008).

The study protocol was approved by the Ethical Committee for Biomedical Research of the Mohammed V University of Rabat (no. 627; July 2019).

### Tick hosts

For the feeding of different stages of *H. marginatum*—i.e., larva, nymph and adult—adult male New Zealand white rabbits of 1.5–2 kg were kept in individual cages. Water was given ad libitum and food as per the recommendation of the food manufacturer. For the feeding of adults, female sheep of > 6 months old and weighing > 20 kg were used. Sheep were examined to be free from hemo-parasites based on a blood smear and were accommodated in individual masonry pens (130 × 80 cm), located in a closed shed with ventilation and slatted floor without contact with other animals; hay and water was provided without restriction.

### Tick feeding

Before tick infestation, hosts were examined and checked to be healthy and free of ticks. Animals re-infested by ticks have a state of local hypersensitivity which causes tick detachment. To avoid tick losses due to this host immune reaction, animals were infested with ticks only once. Hosts were infested at the ears that were shorn after disinfection and the ear canal was protected by cotton before tick application. A cotton bag of 15 × 7 cm for rabbit and 25 × 10 cm for sheep was used for tick infestation. Bags were open at both ends. One side was attached to the base of the animal ear with an adhesive bandage. Ticks were put on the ear from the other side of the bag, and next the bag was folded and closed using an adhesive bandage. Tubes containing ticks were placed on ice for 1–2 min, then picked up by a watercolour paintbrush (around 100 unfed larvae or 30 nymphs) and put inside the tick-feeding bag on the rabbit ear. Unfed adults (15 males and 15 females) were put in a bag and placed tightly around the rabbit/sheep ear to prevent tick escape. The bag was opened and checked daily to prevent ear ischemia and oedema around the pinna and to harvest engorged ticks using a soft forceps. During the tick feeding cycle, animals were maintained in daily recorded ambient temperature (25–28 °C) and relative humidity (RH ≥ 75%).

### Tick rearing

Ticks collected in the field were put in desiccators to maintain 84% RH using potassium hydroxide salt and distilled water (Winston and Bates 1960). Desiccators were kept in an incubator to hold ticks at 28 °C for oviposition. When the oviposition was completed, the dead females were removed from the tube to avoid fungal growth on the dead ticks and subsequent contamination of the eggs. The freshly laid eggs were aliquoted in separate tubes (100 mg/tube amounting to approximately 2000 larvae/tube) and kept under the same conditions of temperature (28 °C) and RH (84%) until hatching. This collection of hatched larvae was the starting point of the study.

In a first experiment, hatched larvae from one engorged female were split into groups of 200 and each group was assigned to a rabbit (in three replicates, i.e., three rabbits). The larvae fed on the rabbit, but most of them continued moulting to nymphs on the rabbit and also the nymphs continued feeding on the same rabbit. Therefore, we only report time from the unfed larva placement to nymph detachment for the first part of the experiment. Next, collected engorged nymphs were divided into separate groups (20 per tube) until moulting. Moulting was studied at 18 and 28 °C using three replicates of 20 nymphs at each of the two temperatures. After moulting, adults were collected and put on a rabbit and sheep as described before (15 males and 15 females/animal) in three replicates. After detachment of the adults from the host, two homogeneous groups were selected, each with 10 engorged female ticks, washed with distilled water, weighed and kept in individual rearing tubes at the two temperatures to establish the preoviposition and the oviposition periods. Eggs laid by each tick were counted daily and weighed at the end of the oviposition. To assess the incubation period and the percentage hatching, we kept separate batches of 100 eggs laid by each female on the 5th day of oviposition at each temperature (Ouhelli and Pandey 1984).

A second experiment was set up to determine the larval and nymphal feeding and moulting period. Larvae obtained from a second engorged female tick were put on rabbits to feed. We collected some of the fully engorged larvae on the 6th day from the rabbit ears using a small curved forceps. Collected larvae were washed in distilled water, dried by paper towel and divided by batches of 50 per rearing container. The time to moulting was then determined at the two temperatures, in three replicates each.

### Statistical analysis

For each type of event, i.e., moulting or feeding, the required time is summarized in two ways. First, the median of those ticks that have the event in a replicate is derived, and the mean and the standard deviation of the medians of the replicates are then calculated. Second, the median time to the event for all ticks and the percentage success is derived. The effect of temperature on moulting is tested by the Cox proportional hazards model using the log-rank test and Kaplan Meier survival curves are used to depict the success of moulting over time. The feeding period duration for nymph and adult was compared by a t-test using the median feeding time for each replicate as response variable.

### Biosecurity

During our experiments, biosecurity measures were taken to prevent accidents. Handlers were well trained and informed and warned about the danger of tick bites. Personal protection precautions such as the use of special clothes, gloves and cover heads were obligatory during all steps of tick handling. To avoid tick escape into the environment all rooms holding animals were protected by tick traps (double-sided sticky tape, grease and petroleum jelly). In the case of bag detachment, animals were removed from their crates to be checked and rabbits were combed with a metal or plastic flea comb to locate ticks.

## Results

### Time from unfed larva to engorged nymph

The mean (± SD) percentage of larvae that successfully attached to a host and developed to an engorged nymph was equal to 52.3 ± 1.5%. The mean (± SD) time required for the larvae to develop into nymphs was 19.98 ± 0.85 days.

### Development time of separate stages

**Fig. 1:**
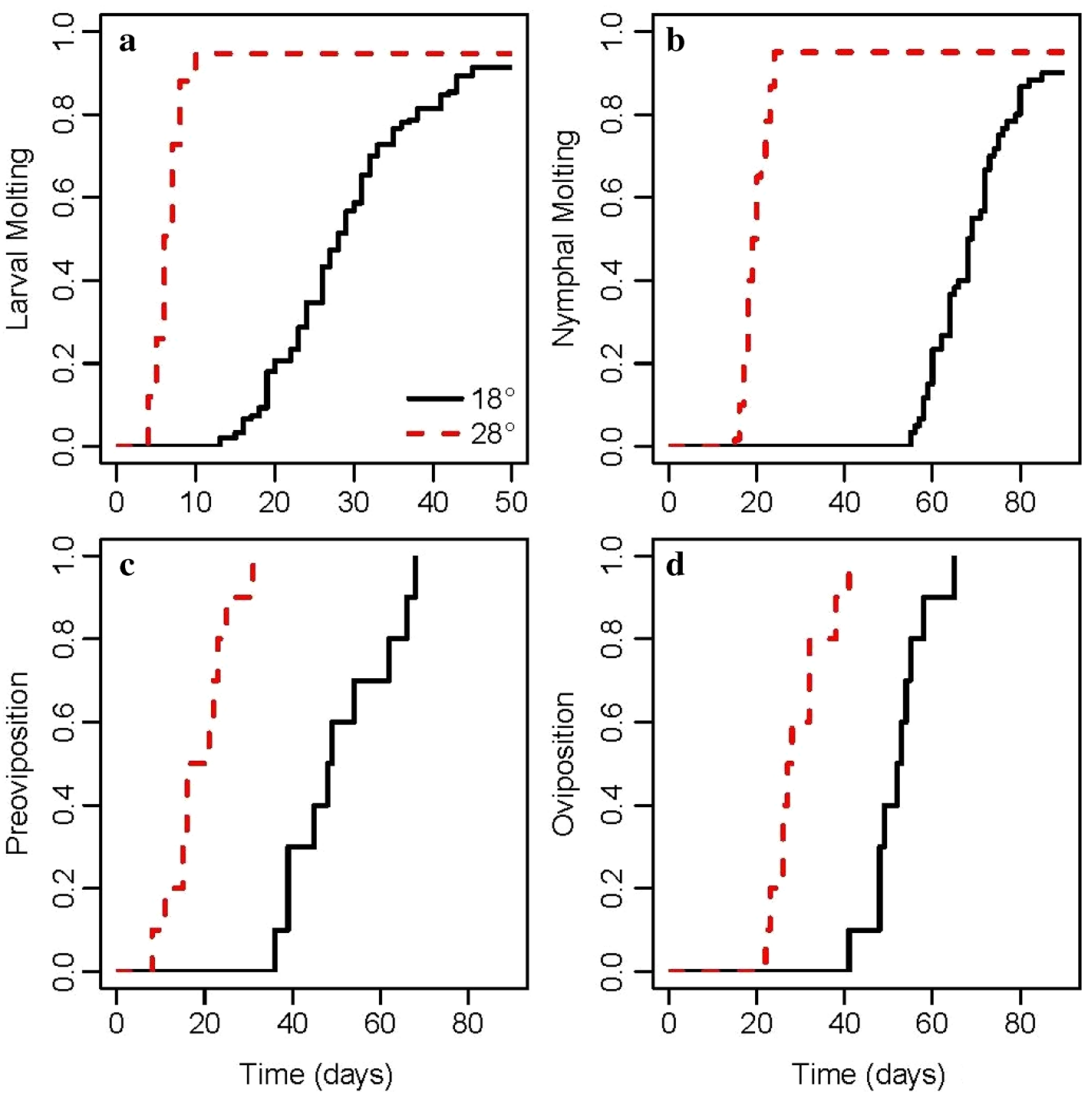
Development time (days) of non-parasitic stages of *Hyalomma marginatum* ticks at 18 and 28 °C: **a** larval molting, **b** nymphal molting, **c** preoviposition period, and **d** oviposition period

Larval moulting occurred faster at 28 °C than at 18 °C, with the hazard ratio equal to 4.54 (95% CI = 3.50–5.89, P < 0.001; Fig. 1a). Most of the larvae moulted to nymph (91% at 18 °C and 95% at 28 °C; Table 1). Nymphal feeding was highly successful, as on average 92.2% engorged, with time to engorgement equal to 9.4 days (Table 1).

**Table 1:** Developmental periods (days) and success (%) of parasitic and non-parasitic stages of *Hyalomma marginatum* reared under laboratory conditions

Stage		Temp (ºC)	Success (%) (SD)	Development (days)	
				Median (range)	Mean (SD)
Larva	Moulting	18	91.3 (7.0)	27.7 (13–45)	27.70 (1.01)
		28	94.7 (5.0)	6.0 (4–10)	6.43 (0.58)
Nymph	Feeding		92.2 (5.85)	8.9 (5–15)	9.42 (0.18)
	Moulting	18	90.0 (5.0)	68.0 (55–85)	67.80 (0.32)
		28	95.0 (5.0)	19.0 (15–24)	19.50 (1.04)
Adult	Feeding		94.4 (9.62)	16.0 (11–21)	16.20 (0.44)
	Preoviposition	18	100	48.5 (36–68)	50.6 (11.57)
		28	100	18.5 (8–31)	18.6 (6.89)
	Oviposition	18	100	52.5 (41–65)	52.3 (6.50)
		28	100	27.5 (22–41)	29.5 (6.22)
	Postoviposition	18	100	2.0 (1–5)	2.4 (1.35)
		28	100	3.0 (2–6)	3.5 (1.27)

Nymphal moulting occurred faster at 28 °C than at 18 °C (hazard ratio 4.86; 95% CI = 3.22–7.35, P < 0.001; Fig. 1b). Almost all nymphs moulted to adults (90% at 18 °C, 95% at 28 °C; Table 1).

None of the adult ticks that were placed on a rabbit to be fed attached; they all dropped off without engorgement. Most of the adults (94.4%) attached on sheep and engorged with time to engorgement equal to 16 days (Table 1). After engorgement adult females weighed 413 ± 19.1 mg (mean ± SD).

The preoviposition period also depended on temperature (Fig. 1c; Table 1). Preoviposition occurred significantly faster at 28 °C than at 18 °C. As there was no overlap between the two Kaplan Maier curves, i.e., each tick at 28 °C had a shorter preoviposition period than any of the ticks at 18 °C, the log rank test could not be executed. Similarly, the oviposition period was shorter at 28 °C than at 18 °C (hazard ratio 38.67; 95% CI = 4.43–337.60, P < 0.001; Fig. 1d; Table 1). The rate of oviposition reached a peak between 8 and 11 days when the ticks were maintained at 18 °C, which was reduced to 5–6 days at 28 °C.

There was no significant difference between 28 and 18 °C for postoviposition period, i.e., time to death after oviposition (hazard ratio equal 0.51; 95% CI = 0.22–1.27, P = 0.15). The number of eggs did not differ between both temperatures (18 °C: 3261 ± 1001; 28 °C: 3465 ± 1402; P = 0.71).

The hatching of eggs was inhibited at 18 °C and thus no larvae could be harvested at this temperature. At 28 °C, the mean (± SD) incubation period of the eggs was 28.9 ± 5.9 days (range 22–38 days). Of all eggs laid, on average 69.8 ± 6.4% hatched, and larvae were ready to feed on animals after 6.9 ± 0.99 days after hatching.

### Time from tick collection in the field to second-generation of larvae

**Fig. 2:**
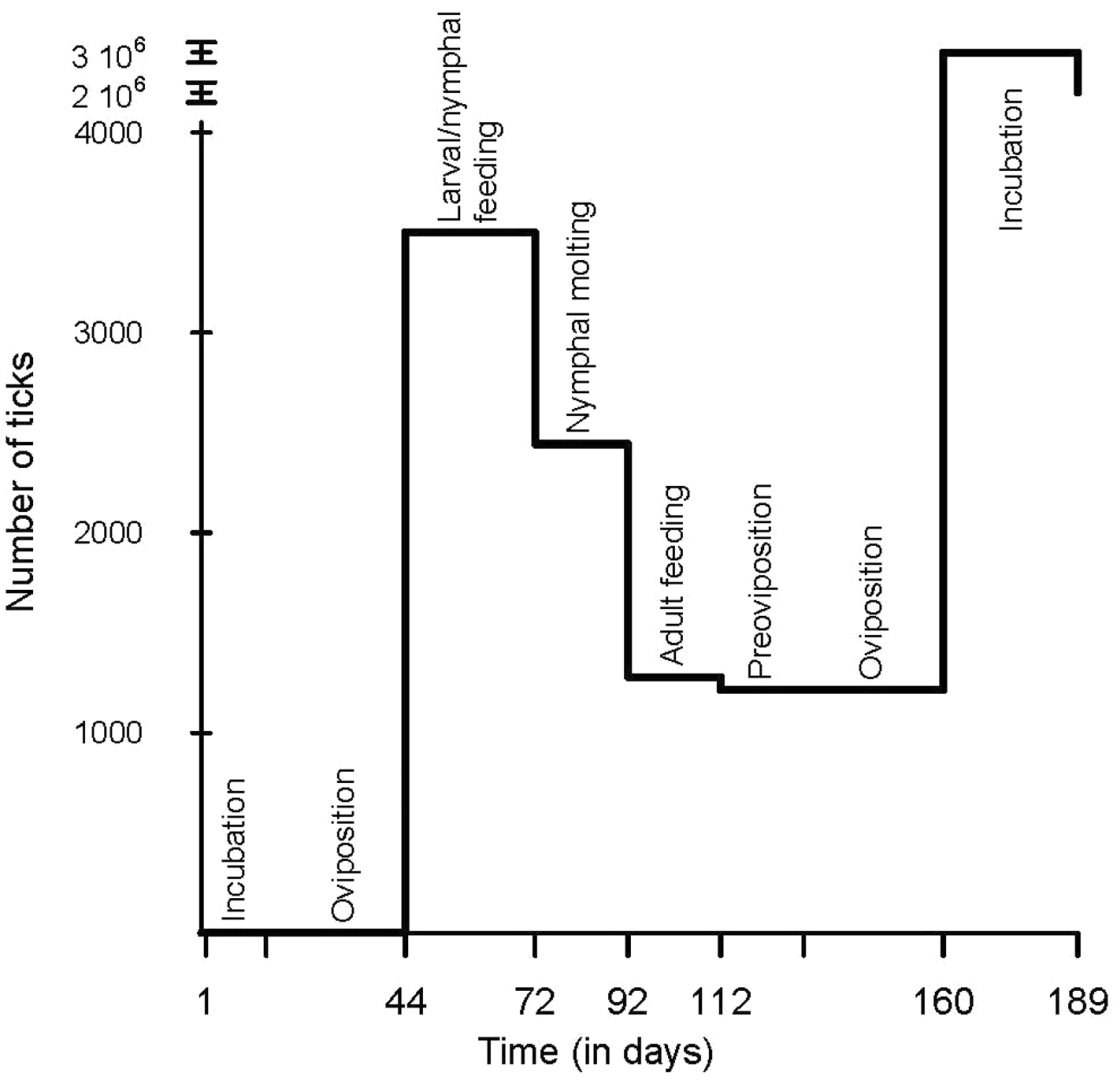
Required time for the development of *Hyalomma marginatum* ticks from field collection to second-generation larvae, with the number of individuals at each stage on the y-axis

Under the conditions of 28 °C and 84% RH, it took 189 days from the collection of an engorged female to the hatching of second-generation larvae, and 103 days to complete one life cycle from unfed larvae of the first generation to freshly hatched larvae of the second generation (Fig. 2). Starting from one engorged field tick, on average 3465 eggs will be obtained. Going through the various stages (see Fig. 2) and taking into consideration all the losses, this will eventually lead on average to 1200 adult ticks and a potential for 2 million second-generation larvae.

## Discussion

In this study, the larval-nymphal feeding period of *H. marginatum* was about 2 days shorter than that of *H. marginatum rufipes* (22.8 ± 0.13 days) as described by Knight et al. (1978). The adult feeding period of *H. marginatum* was longer than the nymphal feeding period (16.2 vs. 9.4 days, P < 0.001). Similarly, *Hyalomma lusitanicum* adults sucked blood on calves significantly longer than nymphs on rabbits (10.1 vs. 7.6 days; Ouhelli and Pandey 1984). The adult *H. marginatum* feeding period was as long as that of *H. rufipes* (16 days; Chen et al. 2012). The larval feeding period could not be studied in detail because ticks remained on the rabbit until moulting to nymph and it was not evident to determine when larvae stopped engorgement and started premoulting.

Usually the use of rabbits as host to feed all tick stages would have been more practical and less costly; however, in our study *H. marginatum* adults could not be fed on rabbits. This differs from other reports on *Hyalomma* ticks (Knight et al. 1978; Alahmed and Kheir 2003; Ahmed et al. 2011; Chen et al. 2012; Gargili et al. 2013). Our finding matches the biological development of *H. marginatum* in nature, as adult ticks feed on large mammals (Apanaskevich and Horak 2008). The inability of the adult tick to feed on a rabbit is probably due to the morphology of the *H. marginatum* hypostome of the adult tick which is large and is inapt for the thin skin of the rabbit (Choukri, unpubl.). It has been reported that the morphologically similar species *H. lusitanicum* occasionally feeds on wild rabbits but causes severe damage during feeding (González et al. 2016).

Larval moulting was a short period in the life cycle of *H. marginatum.* The nymphal moulting period (19.5 ± 1.04 days) was shorter compared to that of *Hyalomma schulzei* (24.3 ± 0.05 days) reared at 28 °C and 75% RH (Al-Asgah 1992). At 28 °C, the preoviposition, oviposition and egg incubation periods of *H. marginatum* were all longer than those of *H. anatolicum* (6.4, 21.8 and 21.7 days, respectively) in a study by Ghosh and Azhahianambi (2007). However, the egg incubation period was similar to *H. marginatum rufipes* (29.3 ± 2.9 days) studied under similar conditions (Knight et al. 1978), but highly reduced compared to the 59.1 ± 0.84 days determined under 20 ± 1 °C and 50% RH (Chen et al. 2012).

Temperature is one of the determining factors in the life cycle of ticks (Snow 1969; Alahmed and Kheir 2003; Ghosh and Azhahianambi 2007; ElGhali and Hassan 2010). Our study confirms that the non-parasitic stage periods (moulting, preoviposition, oviposition and egg incubation) of *H. marginatum* are influenced by temperature, with development occurring faster at 28 °C than at 18 °C, at fixed 84% RH. In our study, eggs did not hatch at 18 °C, indicating no biological development of eggs of this tick at this low temperature. This contrasts with *H. anatolicum* eggs, that did hatch at 18 °C (Ghosh and Azhahianambi 2007). Relative humidity was considered as the limiting factor of the distribution of *H. marginatum* adults and immature stages (Valcárcel et al. 2020), whereas our study showed temperature as a limiting factor for egg development. The failure of *H. marginatum* eggs to hatch at lower temperature could be an important factor explaining why closely related species such as *H. anatolicum* have a different geographical distribution (Vatansever 2017). In the study region, *H. marginatum* seem to be well adapted to the warmer and temperate climate.

We also observed that the viability of the engorged larvae and nymphs was less affected by temperature. Similar to previous reports (Logan et al. 1989; Al-Asgah 1992; Chen et al. 2009), this study showed that the deposited egg number is proportional to its weight and independent of temperature.

*Hyalomma marginatum* behaved invariably as a two-host tick and completed its life cycle in 71–133 days with an average of 103.3 days when non-parasitic stages were incubated at 28 °C and 84% RH. This duration is similar to the 107.4 days reported by Al-Asgah (1992) for *H. schulzei*, of which all parasitic stages were fed on rabbits and ticks were handled at 28 °C and 75% RH. However, this duration is shorter than the 180.2 days for *H. rufipes* (Chen et al. 2012), when immature stages were fed on rabbits and adults were fed on sheep, while non-parasitic stages were maintained at 20 °C and 50% RH.

This study provides the time required from tick collection in the field to the production of second-generation larvae in the laboratory, which is highly relevant information when scheduling experiments requiring a large number of larvae, such as phenotypic and genotypic acaricide resistance tests. It would be useful to further investigate whether different stages, e.g., egg or larva, could be kept in a dormant stage for some time, and reactivated when required in a study. Furthermore, the non-parasitic stages seem to develop well at 28 °C, but further studies should elucidate whether other temperatures might be even better.

## Conclusion

Due to the short larval and nymphal feeding period, the high rate of moulting, oviposition and hatching of *H. marginatum*, a large number of high-quality *Hyalomma* ticks can be produced in a short time. The use of two hosts for feeding *H. marginatum* is highly recommended, considering that adult *H. marginatum* did not feed on rabbits. As *T. annulata* has no trans-ovarian transmission and rabbits are not susceptible to infection with *T. annulata*, feeding of *H. marginatum* immature stages on rabbits and adults on confirmed non-infected sheep ensures the production of theileriosis-free offspring (Ghosh and Azhahianambi 2007). This study indicated that *H. marginatum* eggs do not hatch 18 °C. At 28 °C, both egg hatching and development of the various tick stages of *H. marginatum* go fast and with few losses.

## Data Availability

Available on demand.

## Code availability

Available on demand.
